# Production of Cookies Enriched with Bioactive Compounds through the Partial Replacement of Wheat Flour by Cocoa Bean Shells

**DOI:** 10.3390/foods12030436

**Published:** 2023-01-17

**Authors:** Ingrid Denardi Soares, Marcela Eduarda Marchi Cirilo, Isabela Gayola Junqueira, Fernanda Maria Vanin, Christianne Elisabete da Costa Rodrigues

**Affiliations:** 1 Laboratório de Engenharia de Separações (LES), Departamento de Engenharia de Alimentos (ZEA), Faculdade de Zootecnia e Engenharia de Alimentos (FZEA), Universidade de São Paulo (USP), P.O. Box 23, Pirassununga 13635-900, SP, Brazil; 2 Laboratório de Processamento de Pães e Massas (LaProPaMa), Departamento de Engenharia de Alimentos (ZEA), Faculdade de Zootecnia e Engenharia de Alimentos (FZEA), Universidade de São Paulo (USP), P.O. Box 23, Pirassununga 13635-900, SP, Brazil

**Keywords:** cocoa hull, waste valorization, bioactive compounds, protein functionalities, texture profile, color parameters

## Abstract

Approximately 500 thousand tons of cocoa bean shells (CSs) are generated annually and treated as waste. However, their composition is of great nutritional, technological, and economic interest due to their dietary fiber (46.4 to 60.6%), protein (11.6 to 18.1%), and lipid contents (2 to 18.5%), as well as the presence of flavonoids and alkaloids. Thus, this study aimed to obtain CS flour by milling the CSs, characterizing the flour according to its chemical composition and functionalities, and then applying it in the production of cookies, substituting a wheat flour portion (10, 20, 30, and 40%) with CS flour. Cookies were characterized in terms of water, lipids, proteins, phenolic (PC), and total flavanol (FLA) contents, and specific volume (SV), hardness (H), and L*, a*, and b color scale parameters. Increasing the amount of CS showed positive results, as the cookies were enriched with PC (0.68 to 2.37 mg gallic acid equivalents/g of sample) and FLA (0.10 to 0.19 mg epicatechin equivalents/g of sample) but increased hardness (353 to 472 N). By associating the responses, it was concluded that the wheat flour replacement with 30% CS presented values of PC and FLA 3 and 1.6 times higher than the control and could be a formulation of interest to consumers.

## 1. Introduction

Cocoa bean shells (CSs) are a byproduct of the cocoa industry. They are removed together with the germ before or after roasting [1] and treated as waste. CSs are commonly utilized as fuel for boilers in the processing industry, compost for agricultural use, and animal feed, among other uses [1]. However, this material is of great nutritional, technological, and economic interest because it contains more than 50% dietary fiber and approximately 15% protein, as well as bioactive compounds such as flavanols and alkaloids [2,3].

Cocoa derivatives are widely used in the food industry, being applied as ingredients in many products as they provide flavor characteristics that are highly appreciated by consumers [4]. In addition, the world production of cocoa beans was estimated at more than 4.8 million tons in 2021/2022 [5]. Therefore, since the shell represents approximately 10% of the cocoa bean [6], an annual production of approximately 500 thousand tons of CSs generated during chocolate production can be estimated.

The appreciation of the utility of agricultural wastes is increasing due to the scarcity of natural resources. Thus, there is great interest in applying these materials as food ingredients with added value. Some works that value CSs have been reported [1,4,7]. There are, among others, studies on the application of this material in various food formulations such as extruded snacks, biscuits, and beef burgers [6,8,9], on the recovery of bioactive compounds present in CSs [3,10,11], and on obtaining the fat from this material by different types of extraction [2,12,13].

Regarding the application of CSs in food products, recent studies have been directed toward applying this material for nutritional enrichment. Jozinovic et al. [6] produced corn extrudates enriched with 5, 10, and 15% CSs and concluded that they could be effectively used for the nutritional enrichment of foods. Barisic et al. [14,15] evaluated the addition of CSs to chocolate and reported that the samples became smoother and darker, resulting in positive reactions from consumers. However, the authors indicated that more research is needed to evaluate and better understand this result. Barros et al. [16] developed food bars using CS, soy, and green banana flours. These bars were submitted for sensorial evaluation and were generally approved by the tasters.

Cookies are frequently consumed because they are considered practical and inexpensive food. They are bakery products poor in some essential nutrients, as they are made from wheat flour, sugar, and fat. To increase their nutritional content and functional appeal, one alternative is to add other ingredients.

Handojo et al. [17], Barros et al. [8], and Rojo-Poveda et al. [18] are examples of studies that used CSs in the production of cookies. The authors report that the high protein and fiber contents in CSs characterize the cookies as functional products, besides enriching them with phenolic compounds. However, in addition to the deeper characterization of CS flour that is still needed, no studies have fully reported the effects of substituting wheat flour with CS flour and the extent to which this substitution is technologically feasible.

Therefore, this study produced cookies by partially substituting wheat flour with CS flour, aiming to obtain products enriched in bioactive compounds. The CS flour was extensively characterized before its inclusion in cookies. The cookies were chemically and physically characterized, and an objective function was used to associate the desirable and undesirable attributes, suggesting a percentage of replacement that optimizes the incorporation of CSs in cookies.

The application of CSs flour directly in the food matrix is a possible approach to enriching products with bioactive compounds in a simplified and more economical way when compared to the methods that provide the recovery of these compounds from the solid matrix for later application. Furthermore, from this application, one can benefit from other characteristics of CS flour, such as its composition rich in fibers and proteins. For this reason, it is essential to evaluate the composition and functionalities of the flour, as well as its effects on product characteristics.

## 2. Materials and Methods

### 2.1. Materials

CSs were donated by a cocoa industry (Bahia, Brazil). Margarine (Claybom, Brazil), brown sugar (Santo Antônio, Brazil), refined sugar (União, Brazil), wheat flour (Globo, Brazil), chemical leavener (Dr. Oetker, Brazil), and soybean oil (Liza, Brazil) were all purchased in the local market. The reagents used were n-hexane from Merck (Darmstadt, Germany); sodium hydroxide, acetone, glacial acetic acid, anhydrous sodium carbonate, and hydrochloric acid from Synth (São Paulo, Brazil); sodium dodecyl sulfate, Folin–Ciocalteau reagent, 4-(dimethylamine) cinnamaldehyde (DMAC), and standards of gallic acid and (-)-epicatechin from Sigma-Aldrich (Saint Louis, MO, USA). All reagents had purities ≥ 98.0%.

### 2.2. Cocoa Bean Shell Flour Production and Characterization

#### 2.2.1. Cocoa Bean Shell Flour Production

The CSs, as received, were ground in a mill (Marconi, MA090, Piracicaba, SP, Brazil, https://www.marconi.com.br/produto/245/mill-with-vertical-rotor-fixed-hammers- (accessed on 19 December 2022)) to obtain CS flour. This flour was characterized by particle size distribution using sieves (Tyler series, Wheeling, WV, USA, https://wstyler.com/particle-analysis/test-sieves/wstyler-test-sieves/ (accessed on 19 December 2022)), allowing the calculation of the average particle diameter according to ASAE standards [19].

#### 2.2.2. Proximate Analysis

The CS flour was then characterized in terms of moisture (Ac 2-41, AOCS, [20]) using a forced-air convection oven (Marconi MA035/1, Piracicaba, SP, Brazil, https://www.marconi.com.br/produto/170/drying-oven-with-circulation-air-exchange (accessed on 19 December 2022)), lipid content with a high-temperature solvent extraction system Ankom XT10 Extractor (Macedon, NY, USA, https://www.ankom.com/product-catalog/ankom-xt15-extractor (accessed on 19 December 2022)) (Am 5-04, AOCS, [20]), and total nitrogen content (Ba 4f-00, AOCS, [20]) using Leco, FP-528 (St. Joseph, MI, USA, http://lecoperu.com/wp-content/uploads/2018/04/FP528_209-137.pdf (accessed on 19 December 2022)). To convert the total nitrogen content result into protein content, the obtained value was multiplied by a factor of 6.25 [21]. The CS flour was also evaluated for mycotoxin and heavy metal contamination. Aflatoxins B1, B2, G1, and G2, and ochratoxin A (OTA) were investigated [22]. Concerning heavy metals, the official AOAC method 2006.03 [23] was followed for the determination of cadmium (Cd), chromium (Cr), lead (Pb), and nickel (Ni). The protein content in the wheat flour used in the formulations was also determined.

#### 2.2.3. Total Phenolic and Flavanol Contents

To quantify the total phenolic compound (PC) and total flavanol (FLA) contents, CSs and cookie extracts were obtained by contacting 0.5 g of a solid sample, previously cold defatted with hexane, and 5 mL of an acetone/water/glacial acetic acid solution (70/29.5/0.5, *v*/*v*/*v*) according to the methodology suggested by Hammerstone et al. [24], with adaptations by Ortega et al. [25]. The dispersions were stirred for 5 min, placed in an ultrasonic bath for 10 min, and stirred three more times for 5 min with a rest of 25 min between each stirring. In the sequence, the mixtures were centrifuged, and the supernatant was filtered through a 0.45 µm syringe filter.

The methodology of Folin & Ciocalteu [26] was used to determine the total phenolics using a gallic acid standard for quantification (calibration curve expressed as y = a∙x, where x is the absorbance and y is the concentration of PC expressed as mg of gallic acid equivalents (GAE)/g of sample). Limits of detection (LOD) and quantitation (LOQ) were determined as the ratio of 3 and 10 times the standard deviation of R^2^ of the regression lines, respectively, to the slope of the calibration curve (a) from two experimental determinations [27]. For this, the extracts were diluted in water. The reaction medium was prepared by adding 0.5 mL of diluted extract, 2.0 mL of 7.5% sodium carbonate solution (*w*/*v*), and 2.5 mL of 10% Folin & Ciocalteu solution (*v*/*v*). After mixing, the samples were kept in a dark environment for 2 h. The determinations were performed in a spectrophotometer (UV-1650PC, Shimadzu, Japan, https://speciation.net/Database/Instruments/Shimadzu-Europe/UV1650PC-;i1296 (accessed on 19 December 2022)), reading the absorbance at 760 nm.

For total flavanol content determination, the 4-(dimethylamino) cinnamaldehyde (DMAC) methodology was utilized [10,28], using epicatechin as a standard (calibration curve expressed as y = a∙x, where x is the absorbance and y is the concentration of FLA expressed as mg of epicatechin equivalents (EE)/g of sample). LOD and LOQ were also calculated as previously described. In this case, the extracts were diluted in ethanol. Aliquots of 0.5 mL of diluted extract and 2.5 mL of DMAC solution (HCl: ethanol, 1:9 + 0.1% DMAC) were added to the cuvette, and the reading was performed at 640 nm over time until the maximum absorbance value was obtained.

#### 2.2.4. Protein Functionalities

The CS and wheat flours were evaluated according to their nitrogen solubility indices (NSI) following Morr et al. [29] with modifications [30]. Water or 0.1 M NaCl solution was used as the solvent at a pH of the high solubility of CS proteins, 11.0 [12].

Emulsifying activity (EAI) and stability (ESI) indices were also determined, as described by Pearce and Kinsella [31]. Emulsions were prepared by mixing the aqueous supernatants from the NSI analysis and soybean oil. An aliquot of this emulsion was added to sodium dodecyl sulfate (SDS) solution (0.1%, *w*/*v*) [2]. Absorbance measurements were performed at 500 nm immediately ($EAI_{t0})\;$and after 10 min ($EAI_{t10})$ using a spectrophotometer (UV-1650PC, Shimadzu, Japan, https://speciation.net/Database/Instruments/Shimadzu-Europe/UV1650PC-;i1296 (accessed on 19 December 2022)). Equation (1) presents the calculation used to determine the stability index of the emulsion (ESI).

(1) $$ESI\left(\%\right)=\frac{EAI_{t10}}{EAI_{t0}}\cdot 100$$

where $EAI_{t10}$ is the EAI immediately after emulsion formation and $EAI_{t0}$ is the index measured after 10 min.

The CS and wheat flours were also evaluated according to water (WAC) and oil (OAC) absorption capacities [32]. The solid material and water or soybean oil were mixed at a 1:1 mass ratio and agitated (10 s every 5 min for 30 min). After this procedure, the samples were centrifuged, and the supernatants were removed. Absorption calculations considered the initial and final masses of the solids.

### 2.3. Cookie Production

Cookies were formulated as suggested by Pareyt et al. [33] with modifications used by Chagas et al. [34]. A control formulation with 0% CS flour was used, which consisted of 238.5 g of wheat flour, 86.0 g of refined sugar, 86.0 g of brown sugar, 47.5 g of margarine, 41.0 g of water, and 5.0 g of chemical leavener. Cookies were also produced using CS flour as a partial replacement for the wheat flour in percentages of 0, 10, 20, 30, and 40% (C0, C10, C20, C30, and C40, respectively) according to the initial mass of the wheat flour. The formulations were produced in duplicate.

For preparation, first, a mixer was used to homogenize the margarine and sugars for three minutes (KitchenAid Professional, KPM5 mixer, USA, https://www.kitchenaid.com/countertop-appliances/stand-mixers/tilt-head-stand-mixers/p.value-bundle-artisan-series-5-quart-tilt-head-stand-mixer-with-flex-edge-beater.ksm150feer.html? (accessed on 19 December 2022)). Then, water was added for two minutes of further homogenization. After adding the flours and yeast, the mixture was homogenized for 2.5 min. The dough was portioned into 16 ± 1 g units, and each portion was molded into a circular shape approximately 6 mm thick and 5 cm in diameter. The samples were baked for 7 min at 165 °C in a Programmable Turbo Oven with convection (Prática Technipan, E250, Brazil, https://www.machinio.com/listings/59554621-pratica-e250-prg-in-brazil (accessed on 19 December 2022)).

#### Cookie Characterization

Cookies with wheat and CS flours were characterized in terms of moisture, lipid, and protein contents (according to Section 2.2.2) and total phenolic and flavanol contents (according to Section 2.2.3). NSI values were determined for the control formulation and the formulation with the maximum replacement of wheat flour by CS (according to Section 2.2.4).

All cookie formulations were evaluated after baking in terms of mass and volume (VolScan profile, VSP300, Stable Micro Systems, Godalming, UK, https://www.stablemicrosystems.com/VolscanProfiler.html (accessed on 19 December 2022)) to calculate the specific volume [35]. Diameter and height measurements were also performed with a caliper to calculate the spreading factor (10-50.5, AACC, [36]).

Cookies were analyzed in terms of hardness by using a texturometer (Texture TAXT2i, Stable Micro Systems Ltd., Surrey, UK, https://www.stablemicrosystems.com/TAXTplus.html (accessed on 19 December 2022)). A 25 mm diameter cylindrical probe with a 5 kg load cell was utilized per Bárcenas and Rosell’s methods [37]. Color parameters (CIELab scale, L*, a*, b*) were also evaluated in a colorimeter (Aifos, Hunter Lab, Brazil, https://www.hunterlab.com/en/products/benchtop-spectrophotometers/aeros/ (accessed on 19 December 2022)) according to the characterization performed by Castro et al. [38].

### 2.4. Optimized Objective Function (OF)

A joint analysis among results from chemical characterization (phenolic and flavanol contents) and instrumental methods (coloration and hardness) was performed as a first effort to delimitate the percentage of replacement of wheat flour by CS flour. An optimized objective function (*OF*) was proposed as the ratio between the responses that must be maximized to the responses that must be minimized (Equation (2)) [39]. The contents of *PC* and *FLA* and the specific volume are considered the variables to be maximized. Conversely, the coloration parameter L* and the hardness are variables to be minimized.

(2) $$OF=\frac{\left(PC\cdot FLA\cdot SV\right)}{\left(L^{*}\cdot H\right)}$$

Here, *PC* is the content of total phenolic compounds, *FLA* is the content of total flavanol compounds, *SV* is the specific volume, L* is the color parameter, and *H* is the hardness. These variables were proportionally coded from 1 to 10, with 1 being the lowest value and 10 being the highest value observed for each variable. This coding was performed so that all variables influenced the *OF* by the same order of magnitude.

### 2.5. Statistical Analysis

The results from the characterizations are expressed as the means of at least two determinations. These results were compared by analysis of variance using the Duncan test [40] at a confidence level of 95% with the aid of the SAS^®^ program (Version 9.3, SAS Institute Inc., USA, https://www.sas.com/en_us/home.html, accessed on 19 December 2022)).

## 3. Results and Discussion

### 3.1. Characterization of the CS Flour

The particle size distribution of the CS flour used in the cookies production is shown in Table 1, and the average particle diameter was 303 ± 42 µm.

#### 3.1.1. Chemical Composition of the CS Flour

Table 2 shows the results obtained after investigating the chemical composition of the CS flour and the statistical analysis performed by the Duncan test with a confidence level of 95%. The values obtained for moisture and protein content agreed with results found in the literature, with mean values of 9 and 17.5%, respectively [10,12,41,42]. The lipid content was similar to the values described by Arlorio et al. [41] (6.8 ± 0.3%) and Lecumberri et al. [42] (6.6 ± 0.4%). However, variations related to the origin and growing conditions of cocoa, besides the quality of the shelling process, among other factors, may occur [1]. The shelling step can produce a shell residue with greater or lesser amounts of germs and nibs, which may be responsible for the increase in lipid content, which made the residue even richer in fat [12].

One of the concerns that there has been with the application of CSs in food products is the possible contamination of the material with mycotoxins or heavy metals [43]. Cocoa beans are susceptible to fungal contamination that can cause deteriorative changes in sensory properties. Additionally, fungi give rise to mycotoxins such as aflatoxins [44] and ochratoxin A (OTA) [45]. These studies have reported that OTA is concentrated in the bark and that there may also be aflatoxins B1, B2, G1, and G2 present. OTA has been linked to nephrotoxic, teratogenic, and immunosuppressive activities and is classified as a possible human carcinogen [46]. Similarly, as reported by Copetti et al. [44], aflatoxins can produce hepatotoxic, teratogenic, mutagenic, and carcinogenic effects.

Based on the results presented in Table 2, it was noted that the CS flour did not show aflatoxin contamination. However, OTA was identified at a level of 1.2 ± 0.5 μg/kg. The amounts of OTA or aflatoxins in cocoa beans and products are regulated in a few countries. Limits were set in Brazil by the National Health Surveillance Agency (ANVISA), which took into account children’s high use of cocoa products. These values are 10 μg/kg in cocoa beans and 5 µg/kg in cocoa products sold in Brazil, for both ochratoxin A and total aflatoxins [47]. However, the European Commission has discarded the need to establish a maximum limit for these mycotoxins in these products [48]. Based on the contents of aflatoxins and ochratoxin reported in Table 2, it is possible to infer that using this raw material as a food matrix would not pose a risk of food poisoning caused by mycotoxins.

Regarding CS contamination with heavy metals, studies have reported that the metals most frequently found in cocoa products are nickel (Ni), cadmium (Cd), chromium (Cr), and lead (Pb). The factors that favor contamination by heavy metals may be using fertilizers, pesticides, and insecticides and contacting metal equipment during fermentation and drying [43]. CSs may have higher compositions of heavy metals due to their high adsorption capacity, which was evaluated in studies that proposed using CSs as a new adsorbent to remove heavy metals [4]. As with mycotoxins, heavy metals are also associated with health problems. Consumption of nickel in high concentrations can result in accumulation in organs such as lungs and kidneys and is also linked to some types of cancer such as throat, stomach, and lung [49]. Excessive lead intake causes lead poisoning with symptoms that include anemia, weight loss, or cognitive impairment [50]. Cadmium, in the form of free ions, interferes with metabolic cycles, such as the transformation of thiamine and proteins, and, in the case of chronic intoxication, also with the metabolism of calcium and phosphorus compounds [51]. Chromium acts as a mutagenic, teratogenic, and carcinogenic substance [52].

In Brazil, ANVISA published a Normative Instruction in March 2021, updating the maximum tolerated limits of contaminants relevant to consumers’ health. The cadmium and lead contents allowed in chocolates and cocoa-based products are 0.3 and 0.4 mg/kg, respectively, for products with more than 40% cocoa. For products with less than 40% cocoa [47], the level is 0.2 mg/kg for both metals. Therefore, CS flour complies with current legislation regarding these heavy metals. No laws were found that established contamination limits for chromium and nickel, but these values were determined in some studies that reported chromium contents of 0.4 to 1.3 mg/kg for cocoa powder and chocolate [53] and nickel contents from 2.3 to 7.6 mg/kg for cocoa beans with shells [54].

#### 3.1.2. Total Phenolic and Flavanol Contents

One of the factors of interest in the use of CS is its composition containing bioactive compounds. The main bioactive compounds in CS are flavanols (epicatechins, catechins, and procyanidins) and alkaloids (theobromine and caffeine) [2,10,55]. The presence of these bioactive compounds provides a good antioxidant potential for CSs, which was evaluated by Okiyama et al. [10]. The consumption of these compounds is related to preventing diseases associated with oxidative stress [56]. Furthermore, these compounds are also valued due to their antimicrobial activity in products [57].

Regarding the Folin & Ciocalteu [26] method, used to determine the total phenolics using a gallic acid standard for quantification, an average calibration curve (y = 12.76862∙x) with high linearity, R^2^ > 0.99963, was obtained. LOD and LOQ values were 1.12 µg of gallic acid equivalents (GAE)/g of sample and 3.75 µg of gallic acid equivalents (GAE)/g of sample.

For total flavanol content determination using the DMAC method with epicatechin as the standard, an average calibration curve (y = 74.51270x, R^2^ > 0.9991) was obtained. The following values were calculated for LOD and LOQ, 0.27 µg of epicatechin equivalents (EE)/g of sample and 0.90 µg of epicatechin equivalents (EE)/g of sample, respectively. The determined LOQ values were lower than the lowest point of calibration curves.

Thus, in this work, the contents of PC and FLA in the CS flour were evaluated, and the values obtained were 10.8 ± 0.1 (mg GAE/g sample) and 1.38 ± 0.05 (mg EE/g sample), respectively (Table 2). These values agree with those reported by Delgado-Ospina et al. [55] for CSs.

Important to mention that the methodologies of Folin–Ciocalteu and 4-dimethylaminocinnamaldehyde (DMAC) used to quantify phenolic compounds and flavanols, respectively, were previously evaluated and validated by Ma et al. [58]. Reproducibility was evaluated based on the relative standard deviations, and both methods showed low values, indicating low variation between measurements made under the same conditions; that is, high precision.

#### 3.1.3. Protein Functionalities

The solubilities of the proteins present in the CSs were similar in water and saline solution, with values of 38 ± 1 and 40 ± 5%, respectively (*p* > 0.05). These values are similar to those reported by Soares et al. [2], who associated this behavior with the composition of the protein fraction, considering that albumins are soluble in water, while globulins are soluble in saline solutions. Bonvehí and Coll [59] suggested that albumins and globulins represent 79% of the protein fraction of CS.

The WAC and OAC represent the ability of a protein to retain their respective liquids, and these factors greatly influence the sensory and technological properties of food products [60]. Water absorption by proteins helps reduce moisture loss in baked products. In contrast, oil absorption helps retain and enhance flavor in food formulations such as sausages, cake mixes, mayonnaise, and salad dressings [61].

The WAC and OAC values obtained for CS flour in this study (5.6 ± 0.4 g of water/g sample and 2.0 ± 0.4 g of oil/g sample), respectively, are similar to those reported by Soares et al. [2], Botella-Martínez et al. [9], and Delgado-Ospina et al. [55] for CS (3.30 to 5.07 g water/g sample) and (1.28 to 2.74 g oil/g sample). Interestingly, CS flour has a higher WAC than wheat flour (0.86 ± 0.01 g of water/g sample).

The CS flour emulsifying capacity was evaluated by determining the EAI, and its stability was evaluated by determining the ESI. CS flour had an EAI of 60 ± 1 m^2^/g with an ESI of 81 ± 1%, which indicates that after 10 min, 81% of the emulsion persisted. The EAI value found here is lower than that reported by Soares et al. [2] for CS (111 ± 6 m^2^/g) and similar to the lowest values reported by Cvitanovit et al. [62] at approximately 75 m^2^/g. It is worth mentioning that these results are very dependent on the batch of material used, which depends on the great variability in composition associated with agro-industrial residues, as discussed for the observed variation in the CS lipid content.

### 3.2. Characterization of the Cookies

**Figure 1:**
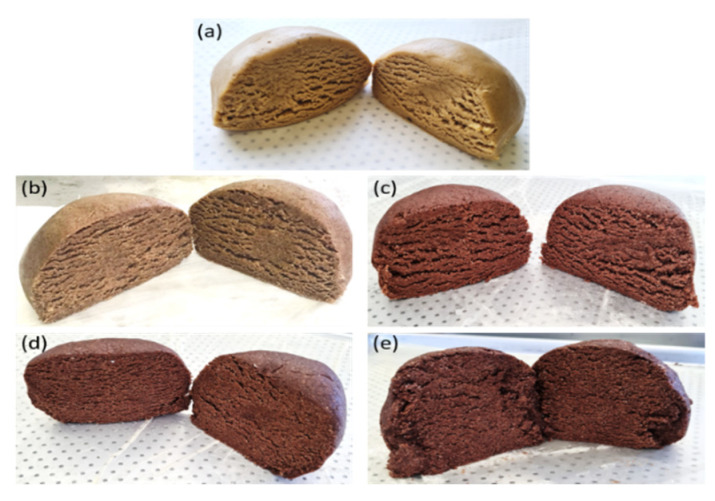
Cookie doughs obtained from formulations with (**a**) 0%, (**b**) 10%, (**c**) 20%, (**d**) 30%, and (**e**) 40% CS flour substituted for wheat flour.

Cookies were formulated by replacing 0, 10, 20, 30, and 40% of the total mass of wheat flour with CS flour. Cookies could be formulated with up to 40% CS flour (*m*/*m*). The dough became heterogeneous and brittle with more CS flour, making it challenging to form cookies. Figure 1 presents images of the doughs formed for each evaluated condition. Wheat flour is essential for the formation of viscoelastic dough due to the presence of glutenin and gliadin in gluten [63]. Thus, the viscoelastic characteristics of the cookies were affected by the reduction in wheat flour percentage, as the contents of these proteins in the dough were lower.

#### 3.2.1. Chemical Composition of the Cookies

Cookies were characterized by their composition in terms of moisture, lipids, proteins, PC, and FLA (Table 2). Considering all the ingredients and their contents from the formulation control (C0) without adding CSs (238.5 g of wheat flour, 86.0 g of refined sugar, 86.0 g of brown sugar, 47.5 g of margarine, 41.0 g of water, and 5.0 g of chemical leavener), and the partial contents of wheat flour that were replaced by CS flour (0, 10, 20, 30, and 40%), it was possible calculating the total percentage of CS flour applied in each formulation. C0, C10, C20, C30, and C40 presented the following percentage values of CS flour 0, 4.7, 9.5, 14.2, and 18.9%. Concerning moisture, there were no significant differences as the percentage of wheat flour replaced by CS flour increased (*p* > 0.05). Chagas et al. [34] evaluated the partial substitution of wheat flour for camu-camu flour in cookies and observed the previously described behavior for product moisture. Rojo-Poveda et al. [18] also evaluated the production of cookies using CS as a partial substitute for wheat flour. These authors reported that the moisture and water activity of the cookies increased due to the higher percentage of CSs added to the formulation. They associated the increased humidity of the formulated cookies with the high concentration of fibers in the flours that replaced wheat flour, which caused greater water retention. According to the nutritional information from the supplier, the amount of fiber in the wheat flour used was 3.2%. In contrast, previous studies report that CSs have a dietary fiber content of approximately 50%, with around 40% being insoluble fiber [2,12].

In terms of the lipid content, it was noted that there is a significant increase from the C0 formulation to the C40 formulation (*p* < 0.05, Table 2). According to previous work, the major fatty acids that compose the cocoa bean shell fat are oleic acid (35.9 mass %), stearic acid (28.6 mass %), palmitic acid (26.4 mass %), and linoleic acid (6.7 mass %), yielding an iodine value of 45 with around 10 mass % of free fatty acids [2]. Proteins are an essential macronutrient of the human diet with several metabolic and physiological functions, such as appetite regulation, weight, and body composition [64]. From a technological point of view, proteins are widely used due to their functional properties, such as WAC and OAC, which can help in the structure of products [6,8]. Regarding the cookie samples, the protein content increased from (9.5 ± 0.4) mass% to (10.7 ± 0.5) mass% when CS flour was incorporated. Barros et al. [8] evaluated the production of wheat flour cookies enriched with a mixture of CS, soy, and green banana flours. The protein content of the formulation containing only wheat flour (200 g) and CS flour (80 g) was (8.6 ± 0.2)%, a value similar to that presented in this work. The authors stated that their cookies can be considered a source of protein. This statement follows Resolution no 54 of 2012 from the National Health Surveillance Agency (ANVISA), Brazil, which defines that a portion of food can be considered a source of protein if it has at least 6 g of protein per 100 g of food [65]. Therefore, the same can be stated for the cookies in this work.

Regarding the determination of total phenolic and flavanol contents (Table 2), the C0 formulation of the cookies presented 0.68 ± 0.04 mg GAE/g sample and 0.105 ± 0.004 mg EE/g sample. For the other formulations, increases in the PC and FLA contents were observed with an increase in the amount of CS flour used.

Barros et al. [8] evaluated the PC contents in cookies made with wheat flour enriched with a mixture of CS, soy, and green banana flour. The authors obtained products with compositions ranging from 0.68 to 1.90 mg GAE/g of sample, with the highest value obtained with the formulation enriched only with CS. Therefore, the increases in the contents of these compounds in the samples were directly related to the increase in the concentration of CS in the formulations. In the present work, the PC contents ranged from 1.5 to 3.4 mg GAE/g for cookies enriched with CS flour.

#### 3.2.2. Physical Properties of the Cookies

**Figure 2:**
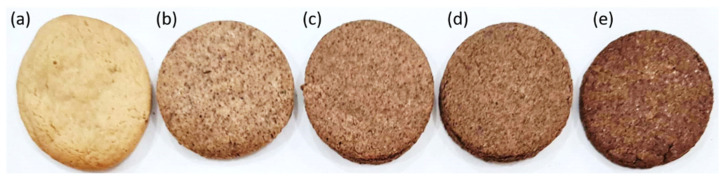
Coloring of the cookies obtained from the formulations with (**a**) 0%, (**b**) 10%, (**c**) 20%, (**d**) 30%, and (**e**) 40% substitution of wheat flour for CS flour.

The cookies were also evaluated for their color, specific volume, spreading factor, and hardness, as shown in Table 3. The coloration of the cookies was evaluated using color parameters from the CIELAB color scale. This scale is composed of the parameter L*, which measures the brightness of the sample on a scale from black to white (0 to 100); a*, which indicates degrees of redness with positive values or greenness with negative values; and b*, which indicates yellowish colors with positive values or bluish with negative values. When evaluating the results (Table 3), it was observed that the luminosity (L*) decreased significantly with increasing concentrations of CS flour in the cookie formulations. This effect can be explained by the darker coloration of CS flour compared to wheat flour. The coloration of CS flour is similar to that of cocoa powder, and consequently, the cookies produced have the appearance of a “chocolate-flavored” product, as seen in Figure 2.

The b* parameter also showed a reduction with an increase in the amount of CS flour added, indicating that the cookies became less yellow. The most remarkable difference observed involved the control formulation, which presented a value of 27.0 ± 0.5, and the formulations supplemented with CS flour presented values ranging from 13.8 ± 0.7 to 18 ± 1. In contrast, the a* parameter increased when the amount of CS flour added increased, indicating that the cookies became redder than the control.

The same behavior was observed by Barros et al. [8] during the production of cookies with CSs. The authors reported that CSs promoted higher L* values than other flours (soybean and green banana) and that as the CS concentration applied became higher, the cookies produced became darker, opaquer, and browner.

Rojo-Poveda et al. [18] also evaluated the coloration of cookies produced with different percentages of CS flour by the CIELAB scale. The authors reported that the parameter that showed the most variation was L*, which decreased when the percentage of CS applied increased, as the cookies became darker due to the brown color provided by the CS flour. In this case, the a* and b* parameters also decreased when the CS content increased, meaning that the cookies became less red and less yellow.

For a better evaluation of the a* and b* parameters, the qualitative attribute of color, hue angle (°Hue), was calculated. Graphically, the 0° angle is considered red, the 90° angle is yellow, the 180° angle is green, and the 270° angle is blue [66]. °Hue values for cookies (Table 3) range from 75 to 59°. The highest calculated value was found for the C0 formulation, and the values tended to decrease as a higher concentration of CS flour was added, which indicates that the cookies went from having yellowish tones to having reddish tones. Barros et al. [8] reported a °Hue of 61.8 ± 0.5 for CS-enriched cookies, a value close to those obtained in this study.

The substitution of wheat flour for CS flour did not influence the spreading factor (*p* > 0.05). Chagas et al. [34] also evaluated the spreading factor of cookies made with wheat flour and camu-camu flour and observed the same behavior. The authors reported that even with a reduction in the amount of wheat flour in the formulation, the spreading factor of the cookies did not vary, which was possibly due to the water retention capacity of the camu-camu flour. The same analogy can be used for CS flour, as the WAC (Table 2) of CS (5.6 ± 0.4 g of water/g of sample) is greater than that of wheat flour (0.86 ± 0.01 g of water/g of sample).

In contrast, Rojo-Poveda et al. [18] reported that the spreading factor of the control formulation (0% CS) was higher than that of cookies with 10 and 20% CS. The authors explained that the reduction in spreading was due to the dilution of the gluten network by the added fiber. A higher fiber content absorbs part of the water, which will no longer be available for the development of the gluten network, which translates into less cookie spreading. In addition to the dilution of the gluten network, the added fiber can also cause other implications, such as the discontinuity of the protein structure that affects the ability of the dough to expand and retain gas [67].

Regarding the specific volume, it was noted that, generally, substituting wheat flour for CS flour causes a significant reduction in this measure. The higher the CS flour concentration employed, the more significant the reduction. The reduction in the cookies’ specific volume with wheat flour replacement can be explained by the reduction in the gluten matrix in the formulations. The gluten matrix reduction decreases the dough’s viscoelasticity and leads to less dough expansion during baking. The same behavior was observed by Castro et al. [38] and Chagas et al. [34] during the production of cookies with orange and camu-camu flours.

The results in Table 3 show that the increase in the concentration of CS flour also caused an increase in cookie hardness. As reported for the reduction in specific volume, the increase in the hardness of the cookies with the increase in the concentration of CS flour can also be explained by the reduction in the gluten matrix, which comes from wheat flour [34]. Another possible explanation is related to the water-absorbing components, such as fibers and proteins, which can reduce the extensibility and increase the hardness of the dough [68].

The same behavior was observed by Handojo et al. [17], Rojo-Poveda et al. [18], and Barros et al. [8] for CS-enriched cookies. These works associated the increase in hardness with the high fiber content of CS flour, which absorbs water, impairing the development of gluten and, therefore, providing harder and less crunchy products. Castro et al. [38] and Chagas et al. [34] observed that replacing wheat flour with other flours resulted in harder cookies, and affiliated this behavior to reducing the gluten matrix in the formulations.

#### 3.2.3. Optimized Objective Function (OF)

**Figure 3:**
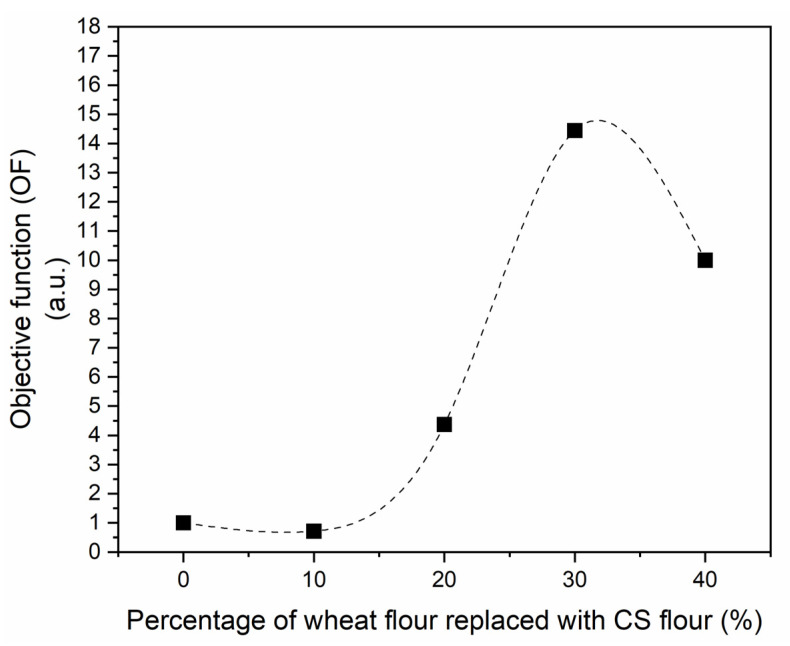
Objective function (OF) in arbitrary units (a.u.) as a function of the percentage of wheat flour replaced with CS flour in cookies: (■) experimental data; (- - -) spline fitting.

As previously mentioned, an optimized objective function (OF, Equation (2)) was proposed as a first effort to delimitate the percentage of replacement of wheat flour by CS flour. Figure 3 shows the OF as a function of the percentage of wheat flour replaced by CS flour.

It can be seen that from formulation C30 onward, undesirable variables (color parameter L*, and the hardness H) begin to exert a more significant influence on the sample, causing a drop in OF. Therefore, it is inferred that the C30 formulation presents the best balance between desirable and undesirable variables. The C30 formulation presents a phenolic compound content of 2.1 ± 0.1 mg GAE/g sample and flavanol content of 0.170 ± 0.005 mg EE/g sample, values 3 and 1.6 times higher than the control (C0 formulation).

This analysis taking into account instrumental texture results, is a first fetch, not intending to substitute the sensory analysis. According to Guiné [69], instrumental and sensorial analyses present benefits and constraints, both highly relevant approaches for bakery products.

## 4. Conclusions

The evaluation of the CS flour regarding its chemical composition and functionalities showed that this material has the potential for application in systems that benefit from these properties, such as bakery products, due to its high water absorption capacity. Increasing the concentration of CS flour applied in the formulations made the cookies darker, indicating a “chocolate flavor” product, and enriched the formulations with flavanol and phenolic compounds. However, a limit for this substitution was observed due to the reduction of the gluten matrix, which plays a fundamental role in bakery products. The main factors that limited the substitution of wheat flour for CS flour to 40% were the loss of the ability to form a homogeneous dough and the increase in the hardness of the cookies.

By associating the desirable and undesirable effects of wheat flour replacement with CS flour through an objective function, it was concluded that the formulation with 30% CS substitution showed the best balance among the desirable (enrichment with phenolics and flavanols) and undesirable (hardness) effects, and could be a formulation of interest to consumers. C30 formulation presents 14.2% of CS flour, with values of total phenolic and flavanol contents 3 and 1.6 times higher than the control formulation.

## Figures and Tables

**Table 1:** Particle size distribution of CS flour.

di (µm)	di + 1 (µm)	CS Flour Mass (g)	Frequency (%)	Average Particle Size (µm)
1680	2280	0.74 ± 0.04	2.9 ± 0.2	303 ± 42
1190	1680	0.78 ± 0.07	2.9 ± 0.3	
841	1190	2.06 ± 0.03	8.04 ± 0.05	
595	841	2.5 ± 0.3	10 ± 1	
420	595	8.2 ± 0.6	32 ± 3	
297	420	5 ± 1	19 ± 5	
<297	297	6 ± 2	25 ± 6	

**Table 2:** Characterization of wheat flour, CS flour, and the cookies produced.

Characterization	Wheat Flour	CS Flour	Cookie Formulation				
			C0	C10	C20	C30	C40
Moisture (mass %)	n.d.	7.6 ± 0.1 B	7.7 ± 0.4 B	8.5 ± 0.3 A	8.6 ± 0.3 A	8.6 ± 0.3 A	8.3 ± 0.3 A
Lipid ^a^ (mass %)	n.d.	6.6 ± 0.3 BCD	6.4 ± 0.3 CD	6.0 ± 0.5 D	7.1 ± 0.3 BC	7.3 ± 0.5 B	8.3 ± 0.3 A
Proteins ^a,b^ (mass %)	15.43 ± 0.08 A	16.0 ± 0.4 A	9.5 ± 0.4 C	10.1 ± 0.4 BC	9.9 ± 0.7 BC	9.8 ± 0.1 BC	10.7 ± 0.5 B
PC (mg GAE/g sample)	n.d.	10.8 ± 0.1 A	0.68 ± 0.04 F	1.05 ± 0.05 E	1.44 ± 0.08 D	2.1 ± 0.1 C	2.37 ± 0.07 B
FLA (mg EE/g sample)	n.d.	1.38 ± 0.05 A	0.105 ± 0.004 C	0.121 ± 0.003 C	0.13 ± 0.01 C	0.170 ± 0.005 B	0.19 ± 0.01 B
Aflatoxins (B1, B2, G1, and G2)	n.d.	<LOQ ^c^	n.d.	n.d.	n.d.	n.d.	n.d.
Ochratoxin A (OTA) (μg/kg)	n.d.	1.2 ± 0.5	n.d.	n.d.	n.d.	n.d.	n.d.
Cadmium (Cd) (mg/kg)	n.d.	0.177 ± 0.001	n.d.	n.d.	n.d.	n.d.	n.d.
Chromium (Cr) (mg/kg)	n.d.	2.2 ± 0.1	n.d.	n.d.	n.d.	n.d.	n.d.
Nickel (Ni) (mg/kg)	n.d.	7.89 ± 0.05	n.d.	n.d.	n.d.	n.d.	n.d.
Lead (Pb) (mg/kg)	n.d.	0.355 ± 0.001	n.d.	n.d.	n.d.	n.d.	n.d.
NSI (%) in water	87 ± 4 A	38 ± 1 B	n.d.	n.d.	n.d.	n.d.	n.d.
NSI (%) in 0.1 M NaCl solution	86 ± 5 A	40 ± 5 B	n.d.	n.d.	n.d.	n.d.	n.d.
WAC (g of water/g sample)	0.86 ± 0.01 B	5.6 ± 0.4 A	n.d.	n.d.	n.d.	n.d.	n.d.
OAC (g of oil/g sample)	0.954 ± 0.001 B	2.0 ± 0.2 A	n.d.	n.d.	n.d.	n.d.	n.d.
EAI (m^2^/g)	n.d.	60 ± 1	n.d.	n.d.	n.d.	n.d.	n.d.
ESI (%) ^d^	n.d.	81 ± 1	n.d.	n.d.	n.d.	n.d.	n.d.

^a^ On dry basis. ^b^ N × 6.25 (AOAC, [21]). ^c^ Limit of quantification of 0.4 µg/kg. ^d^ Equation (1). CS = cocoa shell; PC = total phenolic compound content; FLA = total flavanol compound content; NSI = nitrogen solubility index; WAC = water absorption capacity; OAC = oil absorption capacity; EAI = emulsifying activity index; ESI emulsifying stability index; n.d. = not determined. The same capital letters in the same row indicate differences that were not significant by the Duncan test (*p* ≤ 0.05).

**Table 3:** Physical properties of the cookies produced.

Characterization		Cookie Formulation				
		C0	C10	C20	C30	C40
Coloration	L*	64 ± 1 A	50 ± 1 B	43 ± 1 C	40 ± 1 D	36 ± 2 E
	a*	7.1 ± 0.6 B	7.2 ± 0.2 B	8.4 ± 0.4 A	8.8 ± 0.4 A	9.0 ± 0.5 A
	b*	27.0 ± 0.5 A	18 ± 1 B	16.1 ± 0.6 C	14.8 ± 0.3 D	13.8 ± 0.7 D
	°Hue	75 ± 1 A	67.8 ± 0.8 B	64 ± 1 C	61.6 ± 0.6 D	59 ± 1 E
Spreading factor		5.6 ± 0.7 B	5.9 ± 0.5 AB	6.0 ± 0.4 AB	6.1 ± 0.3 AB	6.7 ± 0.3 A
Specific volume (mL/g)		1.36 ± 0.06 A	1.17 ± 0.05 BC	1.26 ± 0.04 AB	1.19 ± 0.06 BC	1.10 ± 0.07 C
Hardness (N)		353 ± 25 B	432 ± 46 A	452 ± 32 A	451 ± 41 A	472 ± 76 A

°Hue = Hue angle = arctg b*/a*. The same capital letters in the same row indicate differences that were not significant by the Duncan test (*p* ≤ 0.05).

## Data Availability

The data presented in this study are available on request from the corresponding author.
